# Clinical and Laboratory Characteristics of Fabry Disease in Children and Adults: A Focus on Early Kidney Findings

**DOI:** 10.3390/jcm15197456

**Published:** 2026-09-25

**Authors:** Okan Akacı, Sevil Yıldız, Nimet Aktaş

**Affiliations:** 1 Department of Pediatric Nephrology, University of Health Sciences, Bursa Yüksek İhtisas Training and Research Hospital, Bursa 16310, Türkiye; 2 Department of Pediatric Nutrition and Metabolism, University of Health Sciences, Bursa Yüksek İhtisas Training and Research Hospital, Bursa 16310, Türkiye; 3 Department of Nephrology, University of Health Sciences, Bursa Yüksek İhtisas Training and Research Hospital, Bursa 16310, Türkiye; nimetaktas@gmail.com

**Keywords:** Fabry disease, kidney involvement, proteinuria, hypertension, family screening, diagnostic delay

## Abstract

**Background:** Fabry disease may cause clinically silent kidney involvement before marked loss of kidney function. **Objectives:** To characterize pediatric and adult patients with Fabry disease, focusing on early kidney findings and age-related organ involvement. **Methods:** This retrospective, single center study included 16 patients followed between 2016 and 2026. GLA variants were identified by targeted next-generation sequencing. Patients were classified as children (≤18 years, *n* = 7) or adults (>18 years, *n* = 9) at the final evaluation. **Results:** Family screening identified 10 of 16 patients from six families. Six of these ten patients had no complaints before diagnosis. The median age at diagnosis was 24.5 years (range, 7–60). In nine patients with known symptom onset, the median onset age was 14 years (range, 8–35), and the diagnostic delay was 8 years (range, 1–30). At baseline, proteinuria was detected in eight (50%), hematuria in seven (43.8%), and hypertension in three patients (18.8%). Children had no hypertension or marked kidney dysfunction, although proteinuria and hematuria were each observed in two. Among adults, proteinuria occurred in six, microscopic hematuria in five, and hypertension in three. Left ventricular hypertrophy and valvular abnormalities occurred only in adults. At the final evaluation, proteinuria was present in 10 patients (62.5%). One adult experienced an estimated glomerular filtration rate decline from 70 to 14 mL/min/1.73 m^2^ and required kidney replacement therapy. **Conclusions:** The diagnostic delay underscores the value of family screening. At baseline, proteinuria and hematuria were observed in both age groups, whereas hypertension, cardiac abnormalities, and advanced kidney dysfunction were confined to adults. During follow-up, hypertension developed in one pediatric patient. These findings support kidney surveillance from childhood onward.

## 1. Introduction

Fabry disease (FD) is a rare, progressive, X-linked inherited lysosomal storage disorder caused by mutations in the Galactosidase alpha (GLA) gene encoding the enzyme alpha-galactosidase A (α-Gal A). Decreased α-Gal A activity results in the build-up of glycosphingolipids, predominantly globotriaosylceramide (Gb3) and globotriaosylsphingosine (lyso-Gb3), in different cells and tissues. FD is a multisystem disorder in which progressive kidney disease, cardiac involvement, and cerebrovascular events are major contributors to morbidity and premature mortality [1,2]. Although the disease often progresses more acutely in males, clinical manifestations in females exhibit considerable variability owing to differences in X-chromosome inactivation. In both sexes, the phenotypic traits of the condition may differ based on the type of genetic mutation and the remaining enzyme activity [1]. Neuropathic pain, hypohidrosis, gastrointestinal symptoms, angiokeratoma, and corneal verticillata are common features that usually appear during infancy or adolescence, but kidney, cardiac, and cerebrovascular issues may occur at later ages in the classic phenotype. However, milder or organ specific symptoms, particularly in female patients and late-onset phenotypes may be associated with delayed diagnosis [2,3]. In the Fabry Registry data, the median ages at symptom onset and diagnosis were 9 and 23 years for males and 13 and 32 years for females, respectively, suggesting a diagnostic delay of almost 14 years for males and 19 years for females [3].

Kidney involvement is one of the main causes of morbidity and mortality in FD. Glycosphingolipid accumulation can lead to structural and functional impairment in podocytes, glomerular endothelium, mesangial cells, and tubular epithelial cells. Kidney involvement can begin in the early stages with hyperfiltration, microalbuminuria, or proteinuria, and over time, there may be a decrease in estimated glomerular filtration rate (eGFR) and the development of end-stage kidney disease. In individuals with kidney involvement, proteinuria has been reported as one of the strongest clinical indicators of kidney disease progression [4].

Enzyme replacement therapy (ERT) and pharmacological chaperone therapy, when appropriate, are the main treatment options for FD. Starting disease-specific treatment before irreversible kidney, cardiac, or neurological damage develops may be associated with better clinical outcomes. However, the decision to start treatment should be individualized by evaluating the patient’s age, sex, phenotype, genetic variant, symptoms, and the degree of organ involvement together [1,5,6].

Although the general population prevalence of FD is not definitively known, its frequency has been reported to range from 0.17–0.95% in screening studies conducted in high-risk groups, such as those with chronic kidney disease, dialysis, and kidney transplantation [7,8,9]. Due to the rarity of the disease and the variability of clinical findings among patients, the presentation of data from different centers is very important for follow-up and treatment approaches. In addition, data describing age-related patterns of early kidney involvement in pediatric and adult patients, particularly among asymptomatic individuals identified through family screening, remain limited.

In this study, we aimed to describe the demographic, clinical, laboratory, genetic, and biochemical characteristics of pediatric and adult patients with FD followed up at our center, to evaluate systemic involvement by age group, and to emphasize the importance of kidney findings in the early stages of the disease.

## 2. Materials and Methods

### 2.1. Study Design and Participants

This was a retrospective, observational, single-center study of pediatric and adult patients with FD identified at our institution. The study involved 16 patients seen in the Pediatric Metabolism, Pediatric Nephrology, and Adult Nephrology outpatient clinics between 2016 and 2026. Diagnosis was confirmed by molecular genetic analysis. Patients were separated into two groups based on age at the final evaluation: pediatric (≤18 years) and adult (>18 years). This study was reported in accordance with the Strengthening the Reporting of Observational Studies in Epidemiology (STROBE) statement.

### 2.2. Inclusion and Exclusion Criteria

Patients with a molecularly confirmed diagnosis of FD were included. No additional exclusion criteria were applied.

### 2.3. Data Collection and Assessments

Clinical, laboratory, genetic, and imaging data were retrospectively extracted from available medical records and laboratory reports. Results of α-Gal A activity, lyso-Gb3 measurements, and GLA genetic analyses performed at external laboratories were obtained from the available reports. Additionally, audiometric, ophthalmological, and echocardiogram results were recorded. Serum creatinine levels were measured using the Jaffe method traceable to IDMS (CREJ2, Cobas 8000, Roche Diagnostics, Mannheim, Germany). Estimated glomerular filtration rate (eGFR) was calculated using the original Schwartz equation [eGFR = k × height (cm)/serum creatinine (mg/dL)] for pediatric patients, with k = 0.55 for children aged 1–12 years and female adolescents aged 13–18 years and k = 0.70 for male adolescents aged 13–18 years. For adult patients, eGFR was calculated using the 2021 CKD-EPI creatinine equation [10,11]. eGFR categories were defined as G1–G5 according to the KDIGO classification [12]. Urinary protein excretion was assessed separately using available spot urine protein-to-creatinine ratio (UPCR), urine albumin-to-creatinine ratio (UACR), and semi-quantitative dipstick measurements. Quantitative proteinuria was defined as a UPCR > 200 mg/g, whereas albuminuria was defined as a UACR ≥ 30 mg/g [12]. All pediatric participants were older than 2 years (age range, 7–18 years) and were therefore eligible for this UPCR threshold. Dipstick protein results were recorded separately as negative, 1+, 2+, 3+, or 4+. Persistent dipstick protein positivity was defined as protein ≥1+ documented in first morning urine samples at each of three separate clinic visits. Microscopic hematuria was defined as the concurrent presence of ≥1+ blood on a urine dipstick and ≥5 erythrocytes/HPF in a microscopic examination of the urine sediment. Findings attributable to urinary tract infection, urolithiasis, crystalluria, menstruation, or another identifiable cause were not included in the hematuria count. Dipstick proteinuria and hematuria were additionally recorded based on semi-quantitative urine dipstick results. Hypertension (HT) was defined according to the 2017 AAP criteria in pediatric patients and according to the ≥140/90 mmHg threshold in adults [13,14]. The presence of left ventricular hypertrophy was determined based on the assessments documented by pediatric and adult cardiologists in the echocardiography reports. Electrocardiographic abnormalities included a short PR interval, conduction disturbances, or arrhythmias. Valve involvement was defined as the presence of structural abnormalities and/or regurgitation in the mitral, aortic, tricuspid, or pulmonary valves on the initial transthoracic echocardiography.

In the genetic evaluation, the exons and exon-intron boundaries of the GLA gene (NM_000169.3) were analyzed using next-generation sequencing on the DNBSEQ-G99 (MGI, Shenzhen, China) platform following multiplex PCR-based target enrichment. The identified variants were evaluated using the ClinVar, gnomAD, HGMD, and OMIM databases and classified according to the 2015 ACMG/AMP criteria.

α-Gal A activity was measured in dried blood spot (DBS) samples. The assay was performed manually using a fluorometric method with a 4-methylumbelliferyl-containing substrate. Results below the laboratory defined cutoff were reassessed by LC-MS/MS using a Revvity kit. In hemizygous males, the diagnosis was made by detecting low α-Gal A activity in DBS and through genetic confirmation. In heterozygous women, the diagnosis was based solely on molecular genetic confirmation. Due to random X-chromosome inactivation, residual α-Gal A activity in heterozygous women could be at normal or near-normal levels, so enzyme activity measured by DBS was not used as a diagnostic criterion in women [15]. The laboratory-defined reference cutoff was >2 nmol/mL/h. Lyso-Gb3 levels in DBS samples were measured using the liquid chromatography tandem mass spectrometry (LC-MS/MS) method. Analyses were performed using an Agilent 6475 LC-MS/MS system. The laboratory-defined reference interval was 0–6 ng/mL. The same reference interval was applied to male and female patients. Sex-specific reference intervals were not used.

FD was diagnosed based on clinical, biochemical, and genetic data. Phenotypes were classified as classic, late-onset/non-classic, or unknown based on clinical observations, age at symptom onset, enzyme levels in individuals with assessed α-Gal A activity, organ involvement, and the phenotypic features of GLA variants described in the literature. If the findings were inconsistent or insufficient data were available to assign a phenotype, the phenotype was considered unclear.

The clinical severity of FD was evaluated using the Mainz Severity Score Index (MSSI), which consists of general, neurological, cardiovascular, and renal domains [16]. Due to the age-related nature of MSSI-based disease severity measurements and insufficient validation in the pediatric population, the score was calculated only for patients over 18 years of age [17,18]. Raw scores for adult patients were reported without being divided into severity categories. MSSI was calculated based on the clinical status at the last assessment date.

Regarding disease specific treatment, the treatment status, treatment agent used, age at treatment initiation, and treatment duration were recorded. The treatment agents were classified as enzyme replacement therapies (agalsidase alfa and agalsidase beta) and pharmacological chaperone therapy (migalastat). Additionally, treatment changes and any additional therapies administered during follow-up were recorded.

Treatment decisions were determined individually by treating physicians based on a comprehensive assessment of age, sex, phenotype, Fabry disease-related symptoms, biochemical findings, and target organ involvement. In pediatric patients, ERT was initiated in the context of symptoms of Fabry disease and/or signs of early organ involvement, including persistent proteinuria [2,5]. In adults, individuals with Fabry disease-related clinical features or kidney, cardiac or neurological involvement were treated with disease-specific therapy [1,2]. Migalastat was examined only in individuals with a GLA variant considered susceptible to this treatment, taking into account age, kidney function, and acceptability for oral administration [1,2]. The choice between ERT and migalastat was unique and based on variant susceptibility, clinical features, and the treating physician’s judgment.

### 2.4. Statistical Analysis

The data were evaluated using descriptive statistics. Continuous variables were presented as median (minimum-maximum), while categorical variables were presented as counts and percentages. Due to the limited sample size and the inclusion of multiple individuals from the same family in the study, comparative statistical analysis between subgroups was not conducted. No patient was excluded because of incomplete follow-up. Available data from each patient’s initial and last recorded assessments were included in the analysis. Missing data were not imputed. Each variable was analyzed using the available data, and variable specific denominators were reported.

### 2.5. Ethical Considerations

The study protocol was approved by the Medical Sciences Ethics Committee of Bursa Yüksek İhtisas Training and Research Hospital, Health Sciences University (No: 2024-TBEK 2026/08-04, date: 5 August 2026). The ethics committee did not consider informed consent necessary, given the study’s retrospective nature. The study was carried out in compliance with the principles of the Helsinki Declaration.

## 3. Results

A total of 16 patients from six families were included in the study. Each family had one index case; six patients were diagnosed as index cases, while the other ten were identified through screening of family members of the index cases. According to their ages at the time of the study, 7 patients (43.7%) were children, and 9 patients (56.3%) were adults. The median age of the child patients was 14 years (range: 9–18), and 2 of them (28.6%) were male. The median age of the adult patients was 46 years (range: 31–63), with 5 (55.6%) being male. In 9 patients with a known age of symptom onset, the median age of symptom onset was 14 years (range: 8–35), the median age of diagnosis in the entire patient group was 24.5 years (range: 7–60), and the median diagnostic delay in symptomatic patients was 8 years (range: 1–30). The median follow-up period for all patients was calculated as 5.5 years (range: 1–10 years). Of the seven patients who had no complaints before diagnosis, six were identified through family screening. Among the pre-diagnostic clinical features, the most common was acroparesthesia (*n* = 8, 50%), followed by gastrointestinal symptoms (*n* = 5, 31.3%) and ocular findings (*n =* 4, 25%). Auditory or vestibular symptoms, angiokeratoma, and autonomic symptoms were observed in three patients (18.8%), and cerebrovascular events in two patients (12.5%) (Table 1). The index case in Family 4 (patient no 9) was initially asymptomatic. Due to a history of early-onset cardiac disease in distant male relatives, the evaluation revealed low α-Gal A activity and high lyso-Gb3 levels, leading to a diagnosis of FD.

Due to age-related variability in clinical findings and organ involvement in FD, the clinical, laboratory, and imaging findings in the initial evaluation were assessed separately for pediatric and adult patients (Table 2).

In the initial evaluation, proteinuria was detected in 8 patients (50%), hematuria in 7 patients (43.8%), and hypertension in 3 patients (18.8%). In pediatric patients, proteinuria and hematuria were observed in two patients each (28.6%), while hypertension was not detected; in adult patients, proteinuria was present in six (66.7%), hematuria in five (55.6%), and hypertension in three patients (33.3%). In 13 patients (81.3%), the GFR category was G1; in 2 patients (12.5%), it was G2; and in 1 patient (6.3%), it was G3. Among the cardiac findings, valvular disease was the most frequently detected (*n* = 6, 37.5%), left ventricular hypertrophy and electrocardiographic abnormalities were present in four patients (25%), and valvular disease and left ventricular hypertrophy were observed only in adult patients.

The genetic and biochemical characteristics of the patients are shown in Table 3.

Six different GLA variants were identified in six families, and all the variants had been previously reported in the literature. In nine female patients, the variant was heterozygous, while in seven male patients, it was hemizygous. Four of the six variants were classified as pathogenic, one as likely pathogenic, and one as likely benign. Three of the variants were located in exon 3 and were found in a total of eight patients. In the phenotypic evaluation, nine patients (56.3%) had the classic phenotype, four (25.0%) had the late-onset phenotype, and three (18.8%) had an uncertain phenotype. α-Gal A activity was below or at the lower limit of the laboratory reference range in all patients, ranging from 0.1 to 2.0 nmol/mL/hour. In a hemizygous male patient carrying the c.376A>G (p.Ser126Gly) variant, classified as likely benign, the DBS α-Gal A activity was low (1.4 nmol/mL/hour). Although the medical records noted that the α-Gal A activity measured from the patient’s plasma at diagnosis was also low, the previous numerical result could not be retrieved. The DBS lyso-Gb3 level was measured in 14 individuals (baseline values could not be retrieved for two patients) and ranged from 1.0 to 10.0 ng/mL. Four of six male patients had Lyso-Gb3 levels above the standard range compared with only two of eight female patients. In the other six female patients, the readings were within the reference range. The highest lyso-Gb3 level in the patient group (10 ng/mL) was detected in a 9-year-old girl.

The kidney characteristics of the patients in the final evaluations are presented in Table 4.

In the final evaluation, 13 patients remained in the G1 category, 1 in the G2 category, and 1 in the G3 category; a patient who was initially in the G2 category progressed to G5 and required kidney replacement therapy. At baseline and final evaluation, dipstick protein positivity (≥1+) was observed in 8 of 16 patients (50.0%) and 10 of 16 patients (62.5%), respectively. UPCR measurements were available for 12 patients at baseline and 8 patients at final evaluation, with values >200 mg/g in 6 of 12 (50.0%) and 4 of 8 (50.0%), respectively. UACR measurements were available for 10 and 7 patients, with values ≥30 mg/g in 5 of 10 (50.0%) and 4 of 7 (57.1%), respectively. While microscopic hematuria was present in 7 patients (43.8%) at baseline, only 3 adult patients (18.8%) had microscopic hematuria at the final check-up. At the final evaluation, HT was present in six patients (37.5%), including five adults and one pediatric patient. HT developed during follow-up in three patients, including two adults and one pediatric patient. The pediatric patient (patient 13) also developed proteinuria during follow-up. Renin–angiotensin–aldosterone system (RAAS) blockade with an ACEi or ARB was initiated in all 10 patients with proteinuria. At the final evaluation, six patients (37.5%) were taking the prescribed treatment regularly, as reflected in Table 4. The remaining four patients were not using the treatment regularly because of poor adherence.

The median MSSI among nine adult patients was 14 (range: 3–39), with the highest score observed in the patient undergoing kidney replacement therapy.

All nine patients with symptoms before diagnosis received disease-specific therapy. Of the seven patients without complaints before diagnosis, patients 2, 6, and 9 received treatment, whereas patients 5 and 14 declined the recommended treatment. Patient 10 remained under follow-up without disease specific therapy. Patient 13 developed proteinuria and hypertension during follow-up and was initially managed with RAAS blockade. As the proteinuria was not severe and no other significant FD related manifestations were present, ERT was not initiated at this stage. The need for ERT will be reassessed according to subsequent clinical and laboratory findings. Overall, 12 patients received disease specific therapy.

Initial therapy was agalsidase alfa in nine patients and agalsidase beta in three. During follow-up, patient 9 switched from agalsidase beta to migalastat because of difficulty adhering to intravenous therapy. At the final evaluation, nine patients were receiving agalsidase alfa, two were receiving agalsidase beta, and one was receiving migalastat. Among the treated patients, the median age at treatment initiation was 27.5 years (range, 7.5–54 years), and the median treatment duration was 3.75 years (range, 2–10 years).

In Family 2, the index patient carrying the c.376A>G (p.Ser126Gly) variant, classified as likely benign, had persistent proteinuria and joint pain that could not be explained by rheumatological evaluation. ERT was initiated following an integrated assessment of the genetic, biochemical, and clinical findings, and the joint pain improved after treatment. The sibling initially had persistent proteinuria without joint pain but developed joint pain during follow-up, after which ERT was initiated. The paternal family history of early-onset myocardial infarction and heart failure was also considered as part of the individualized clinical assessment. RAAS blockade was prescribed for proteinuria in both siblings but was not used regularly because of poor adherence to oral treatment during adolescence. Kidney biopsy was not performed in either sibling because electron microscopy for the assessment of myeloid or zebra bodies was unavailable at our center. ERT was also recommended for their 61-year-old hemizygous father because he had proteinuria, hypertension and G2 kidney function. However, he declined treatment and remained under clinical follow-up without ERT.

## 4. Discussion

This study examined age-related manifestations of FD in children and adults, with particular emphasis on kidney findings. Fabry nephropathy may begin early in the disease course. Although albuminuria, proteinuria, and a decline in eGFR are commonly used clinical markers, ultrastructural changes in podocytes and tubular epithelial cells may be detected in kidney biopsy specimens by electron microscopy before these abnormalities become evident [19,20]. Since electron microscopy is not routinely used for screening kidney biopsies, clinical evaluation, laboratory tests, and family screening still play a central role in the early diagnosis of kidney involvement. In our study, a significant proportion of patients were diagnosed through family screening, underscoring the importance of this approach. The identification of an index case in six families has enabled the detection of one or more individuals within the same family who had not previously been diagnosed. Similarly, in the TURKFAB study conducted in Turkey, three index cases were identified by screening 313 patients with an unknown cause of chronic kidney disease, and a total of 11 Fabry patients were identified through family screening [8]. For patients with known symptom onset, the median age at onset was 14 years, consistent with the typical emergence of FD manifestations during childhood or adolescence. The median age at diagnosis was 24.5 years, and the median diagnostic delay was 8 years. Because early manifestations are nonspecific and may be evaluated by clinicians from different specialties, diagnosis can be substantially delayed. In our study, the longest diagnostic delay was 30 years. Data from the Fabry Registry have indicated that the time between first symptom and diagnosis is roughly 14 years in men and nearly 19 years in women [3]. Our study showed a shorter median diagnostic delay than previously reported. In addition, 10 of the 16 patients were identified through family screening. Six of these patients had no complaints before diagnosis.

Genetic analysis identified six distinct GLA variants, including five missense variants and one small in-frame deletion. Nine patients were classified as having a classical phenotype, and four as having a late-onset or non-classical phenotype. The phenotypic classification of the three patients from family 2 who carried the c.376A>G (p.Ser126Gly) variant remained uncertain. Published evidence regarding the clinical significance of this variant is inconsistent. In a study evaluating individuals with p.Ser126Gly from five unrelated families, no elevation in Fabry disease-specific biomarkers or evidence of target organ involvement was observed. In addition, a kidney biopsy performed in one patient after 12 years of follow-up showed no findings consistent with Fabry nephropathy. The authors concluded that this variant does not cause Fabry disease on its own [21]. Similarly, p.Ser126Gly was classified as likely benign in a reassessment by Germain et al. involving experts in Fabry disease [22]. Given this uncertainty, treatment decisions in our patients were not based on the genetic finding alone. Genetic classification, familial segregation, sex-specific α-galactosidase A activity, lyso-Gb3 levels, clinical findings, and target organ involvement were considered together [23]. ERT was initiated in the index patient because of persistent proteinuria and joint pain, with improvement in joint pain observed after treatment. The sibling initially had persistent proteinuria without joint pain but subsequently developed joint pain during follow-up, after which ERT was initiated. RAAS blockade was also prescribed for proteinuria but was not used regularly because of poor adherence to oral treatment during adolescence. The 61-year-old male family member remains under follow-up without ERT. Despite these clinical decisions, the phenotypic classification of all three family members was retained as uncertain, and our observations were not considered evidence of the pathogenicity of the p.Ser126Gly variant.

In our study, α-Gal A activity and lyso-Gb3 levels showed different distributions by sex. Low α-Gal A activity in hemizygous males strongly supports the diagnosis. In contrast, in heterozygous women, due to variability in X chromosome inactivation, enzyme activity may be within normal limits. At the same time, while the lyso-Gb3 level was above the reference range in most men, it was within normal limits in most women. These findings are consistent with literature data reporting that α-Gal A activity in women may be normal or near-normal and that a normal lyso-Gb3 level is not sufficient to rule out Fabry disease [24,25,26]. However, the highest lyso-Gb3 level (10 ng/mL) in the study group was measured in a nine-year-old female patient. This finding indicates that the general trend related to sex does not apply to every patient and that lyso-Gb3 levels should be evaluated individually. Therefore, it is important to evaluate molecular findings along with clinical features and target organ involvement in the diagnosis of FD in women.

Clinical findings in our cases, the most frequent symptom before diagnosis was acroparesthesia (8 patients, 50%). Gastrointestinal symptoms (31.3%) and ocular abnormalities (25%) were observed after that. Neuropathic pain and gastrointestinal problems are commonly reported clinical characteristics in the early phases of pediatric FD research. Hopkin et al. studied 352 children and adolescents and found that 59% of boys and 41% of girls had neuropathic pain, and 18% of patients had gastrointestinal symptoms [27]. A Turkish investigation involving 10 patients, mainly adults, found that all patients experienced stomach discomfort and nine patients had neuropathic pain [28]. The increased number of people diagnosed via family screening in our study may have contributed to the decreased frequency of pre-diagnosis symptoms being documented. Furthermore, the retroactive record data may have resulted in underdocumentation of mild or temporary problems.

In our study, proteinuria on the dipstick test was detected in 8 patients initially and in 10 at the final follow-up. In FD, quantitative protein excretion is closely related to the loss of kidney function. In the study by Wanner et al., which evaluated 462 untreated adults, a higher average urine protein/creatinine ratio was associated with a faster eGFR decline and identified as the strongest indicator of kidney disease progression [4]. Although the number of patients with proteinuria increased during the follow-up in our study, these data cannot be considered as an indication of a real increase in protein excretion because most of the measurements were based on dipstick tests. In patients with positive dipstick results, the quantification of albumin or protein excretion should be confirmed using quantitative methods and monitored through serial measurements. Microscopic hematuria was detected in seven patients (43.8%) at baseline and in three patients (18.8%) at the final evaluation. Although alternative causes were excluded, this finding was interpreted descriptively because hematuria is not a dominant or specific feature of Fabry nephropathy.

At baseline and at the final evaluation, the median eGFR values were 122 (range, 55–177) and 120.5 (range, 14–212) mL/min/1.73 m^2^, respectively. Some of the high eGFR values observed in pediatric patients may reflect glomerular hyperfiltration. However, overestimation associated with the original Schwartz equation, particularly at higher GFR levels, cannot be excluded. However, because follow-up durations varied among patients and median values do not reflect individual progress, we did not consider this finding an indicator that kidney function was preserved throughout the follow-up. Indeed, in one male patient, eGFR declined from 70 to 14 mL/min/1.73 m^2^, and kidney replacement therapy was initiated. In the early stages of FD, eGFR can remain normal for a long time, so reliance should not be placed solely on eGFR measurement [29,30]. Schiffmann et al. reported that in patients with significant proteinuria, a baseline eGFR below 60 mL/min/1.73 m^2^ or hypertension is associated with more rapid eGFR loss. These patients should be closely monitored for kidney involvement, and if clinically necessary, kidney biopsy should not be delayed [31]. In the management of FD, especially in patients with hypertension, significant albuminuria/proteinuria, or left ventricular systolic dysfunction, the use of angiotensin-converting enzyme inhibitors (ACEIs) or angiotensin receptor blockers (ARBs) is recommended to achieve RAAS blockade. Warnock et al. evaluated 24 patients receiving ACE-I or ARB in addition to ERT and reported that the annual eGFR loss in patients whose urine protein/creatinine ratio was reduced to the target level was slower than in those who did not reach this target [32]. All 10 patients with identified proteinuria in our study were initiated on ACE-I or ARB therapy to minimize proteinuria and preserve kidney function. However, only six patients regularly take their medicine because of problems with adherence to the treatment. This situation highlights the fact that compliance with the medication is as crucial as the initiation of antiproteinuric therapy in Fabry nephropathy.

Among the characteristic cardiac findings of FD, there are mostly concentric left ventricular hypertrophy, diastolic dysfunction, myocardial fibrosis, conduction and rhythm disturbances, and generally mild valvular anomalies [33]. In our study, left ventricular hypertrophy was detected in 4 of 16 patients (25%), and all were adults. An abnormal electrocardiography finding was identified in four patients (25%), one child and three adults. Valve anomalies were detected in six patients (37.5%), and all these patients were in the adult age group. In the Fabry Outcome Survey data, it has been reported that cardiac hypertrophy appears earlier in men [34]. Studies conducted in children have shown that, although left ventricular hypertrophy is not present, mild structural and functional cardiac changes may occur [35]. Therefore, the presence of a normal echocardiogram does not completely rule out cardiac involvement, especially in male cases. All patients should undergo regular electrocardiography and echocardiography follow-up, and especially in adult patients, cardiac magnetic resonance imaging should be considered to evaluate myocardial accumulation and fibrosis [33].

In our study, auditory or vestibular symptoms associated with transient ischemic attack/ischemic stroke were only detected in adults. However, in a study evaluating 47 FD children, it was reported that six patients developed hearing loss before the age of 18, five of whom had sensorineural hearing loss. Additionally, the children’s hearing thresholds were found to be higher than the healthy reference values [36]. Cerebrovascular events, on the other hand, are quite rare during childhood. In the study by Hopkin et al., which evaluated 352 pediatric patients, no cases of stroke were reported. In contrast, Cabrera-Salazar et al. identified cerebral microvascular changes in two male children aged 8 and 11 years, who were diagnosed through family screening and had no clinical central nervous system involvement, using brain magnetic resonance imaging [27,37]. In our study, the evaluation of the seven children was limited to symptom inquiry and examination findings. The lack of detailed neuroimaging and audiological examination, along with the small number of patients, may have contributed to the inability to detect subclinical findings.

In the study group, pre-diagnosis gastrointestinal symptoms were detected in five patients (31.3%). Complaints such as abdominal pain and diarrhea are among the early signs of Fabry disease that can start in childhood. Gastrointestinal symptoms have been reported in 18% of children in large case series. In another study involving patients from different age groups, they were reported in 52%, with abdominal pain and diarrhea being the most common complaints [27,38]. Due to the retrospective evaluation, mild and intermittent complaints may have been underreported in our study.

In our adult patients, the median MSSI was 14 (range, 3–39), with the highest score of 39 observed in the patient receiving kidney replacement therapy. The broad range of raw scores suggests variability in disease burden, even among related patients. Because raw MSSI scores are influenced by age, the results were interpreted descriptively and were not used for comparisons between pediatric and adult patients [17].

The strengths of our study include the evaluation of pediatric and adult patients in the same center and under the same assessment framework, the detailed monitoring of the family-based diagnostic process, and the patient-specific tracking of kidney findings from the initial to the final evaluation.

There are also some limitations to the study. The limited sample size and the fact that the 16 patients came from only six families may have increased the impact of phenotypic similarities among individuals within the same family on the overall findings. Therefore, no comparative analysis was conducted between age groups, and the findings were presented descriptively. Quantitative UPCR and UACR measurements were not available for all patients at both time points, limiting the assessment of the magnitude and longitudinal course of urinary protein excretion. In addition, Fabry nephropathy was not histologically confirmed because kidney biopsy with electron microscopic evaluation was not performed. The lack of systematic application of cardiac and cranial magnetic resonance imaging may have led to an underestimation of extrarenal involvement. The generalizability of these findings may be limited by the small, single center sample and the inclusion of multiple related individuals from only six families. Confirmation in larger multicenter studies including more diverse FD populations is warranted.

## 5. Conclusions

This study supports the value of family screening in identifying individuals with FD who have not yet been diagnosed. While proteinuria is observed in both age groups, significant kidney function loss and extrarenal organ involvement have been predominantly detected in adults. Therefore, in cases of FD, proteinuria, blood pressure, and eGFR should be regularly monitored from childhood onward. Our findings also indicate that GLA variants with disputed pathogenicity should be evaluated alongside biomarkers and organ findings.

## Figures and Tables

**Table 1:** Demographic Characteristics and Clinical Manifestations Before Diagnosis of Fabry Disease.

Patient No.	Family No.	Age * (Years)	Sex	Diagnostic Route	Symptom Onset Age (Years)	Age at Diagnosis (Years)	Diagnostic Delay (Years)	Acro- Paresthesia	Angio- Keratoma	GI Symptoms	Autonomic Symptoms	Ocular Findings	Auditory/ Vestibular Symptoms	Cerebro- Vascular Event
1	1	46	Female	Family screening	30	38	8	Yes	No	No	No	No	No	No
2	1	12	Female	Family screening		7		No	No	No	No	No	No	No
3	1	17	Male	Index	12	14	2	Yes	No	No	No	No	No	No
4	2	18	Female	Index	11	12	1	Yes	No	Yes	No	No	No	No
5	2	61	Male	Family screening		60		No	No	No	No	No	No	No
6	2	14	Female	Family screening		11		No	No	No	No	No	No	No
7	3	18	Male	Family screening	14	16	2	No	No	Yes	No	No	No	No
8	3	35	Female	Index	20	25	5	Yes	No	No	No	No	No	No
9	4	36	Male	Index		33		No	No	No	No	No	No	No
10	4	31	Male	Family screening		24		No	No	No	No	No	No	No
11	5	39	Male	Family screening	20	30	10	Yes	No	Yes	No	Yes	No	Yes
12	5	63	Female	Index	35	54	19	Yes	Yes	Yes	Yes	Yes	Yes	No
13	6	12	Female	Family screening		10		No	No	No	No	No	No	No
14	6	9	Female	Family screening		7		No	No	No	No	Yes	No	No
15	6	48	Male	Family screening	8	38	30	Yes	Yes	No	Yes	No	Yes	Yes
16	6	46	Female	Index	8	36	28	Yes	Yes	Yes	Yes	Yes	Yes	No

* Age at the last evaluation. Autonomic symptoms include hypohidrosis/anhidrosis and/or heat/cold intolerance. Ocular findings include cornea verticillata and/or Fabry cataract/retinal vascular tortuosity. Auditory/vestibular symptoms include hearing loss, tinnitus, and/or vertigo. Cerebrovascular events include transient ischemic attack and/or ischemic stroke. GI, gastrointestinal.

**Table 2:** Baseline Clinical Findings According to Age Group.

Clinical Manifestation	Pediatric Patients (*n* = 7)	Adult Patients (*n* = 9)	Total (*n* = 16)
Kidney manifestations			
Hypertension	0/7 (0.0)	3/9 (33.3)	3/16 (18.8)
Dipstick proteinuria	2/7 (28.6)	6/9 (66.7)	8/16 (50.0)
Microscopic hematuria	2/7 (28.6)	5/9 (55.6)	7/16 (43.8)
CKD stage G2–G5	0/7 (0.0)	3/9 (33.3)	3/16 (18.8)
Cardiovascular manifestations			
Left ventricular hypertrophy	0/7 (0.0)	4/9 (44.4)	4/16 (25.0)
Abnormal electrocardiogram	1/7 (14.3)	3/9 (33.3)	4/16 (25.0)
Valvular abnormality	0/7 (0.0)	6/9 (66.7)	6/16 (37.5)
Neurological and audiological manifestations			
Acroparesthesia	2/7 (28.6)	6/9 (66.7)	8/16 (50.0)
Transient ischemic attack/ischemic stroke	0/7 (0.0)	2/9 (22.2)	2/16 (12.5)
Auditory/vestibular symptoms	0/7 (0.0)	3/9 (33.3)	3/16 (18.8)
Gastrointestinal manifestations			
Gastrointestinal symptoms	2/7 (28.6)	3/9 (33.3)	5/16 (31.3)
Dermatological, ophthalmological, and autonomic manifestations			
Angiokeratoma	0/7 (0.0)	3/9 (33.3)	3/16 (18.8)
Ocular findings	1/7 (14.3)	3/9 (33.3)	4/16 (25.0)
Autonomic symptoms	0/7 (0.0)	3/9 (33.3)	3/16 (18.8)

Data are presented as n/N (%). Auditory/vestibular symptoms include hearing loss, tinnitus, and/or vertigo. Ocular findings include cornea verticillata and/or Fabry cataract/retinal vascular tortuosity. Autonomic symptoms include hypohidrosis/anhidrosis and/or heat/cold intolerance. CKD, chronic kidney disease.

**Table 3:** Genetic and Biochemical Characteristics of the Fabry Disease Cohort.

Patient No.	Family No.	Sex	GLA Variant (cDNA)	Protein Change	Exon	Zygosity	Phenotype	ACMG Classification	α-Gal A (nmol/mL/h) ^a^	Lyso-Gb3 (ng/mL) ^b^
1	1	F	c.547G>A	p.Gly183Ser	3	Heterozygous	Classic	P	0.9	2.0
2	1	F	c.547G>A	p.Gly183Ser	3	Heterozygous	Classic	P	1.7	1.7
3	1	M	c.547G>A	p.Gly183Ser	3	Hemizygous	Classic	P	0.1	8.0
4	2	F	c.376A>G	p.Ser126Gly	3	Heterozygous	Uncertain	LB	2.0	1.0
5	2	M	c.376A>G	p.Ser126Gly	3	Hemizygous	Uncertain	LB	1.4	2.0
6	2	F	c.376A>G	p.Ser126Gly	3	Heterozygous	Uncertain	LB	1.7	2.0
7	3	M	c.160C>T	p.Leu54Phe	1	Hemizygous	Later-onset	P	0.3	3.8
8	3	F	c.160C>T	p.Leu54Phe	1	Heterozygous	Later-onset	P	0.7	2.0
9	4	M	c.886A>G	p.Met296Val	6	Hemizygous	Later-onset	LP	0.1	7.0
10	4	M	c.886A>G	p.Met296Val	6	Hemizygous	Later-onset	LP	0.1	8.0
11	5	M	c.422C>T	p.Thr141Ile	3	Hemizygous	Classic	P	0.1	8.0
12	5	F	c.422C>T	p.Thr141Ile	3	Heterozygous	Classic	P	1.5	7.0
13	6	F	c.1212_1214delAAG	p.Arg404del	7	Heterozygous	Classic	P	1.6	1.0
14	6	F	c.1212_1214delAAG	p.Arg404del	7	Heterozygous	Classic	P	0.4	10.0
15	6	M	c.1212_1214delAAG	p.Arg404del	7	Hemizygous	Classic	P	1.6	NR
16	6	F	c.1212_1214delAAG	p.Arg404del	7	Heterozygous	Classic	P	0.2	NR

^a^ The laboratory reference value for α-Gal A activity measured from dried blood spot samples was >2 nmol/mL/h for all patients. ^b^ The laboratory reference interval for Lyso-Gb3 measured from dried blood spots was 0–6 ng/mL. α-Gal A, α-galactosidase A; ACMG, American College of Medical Genetics and Genomics; cDNA, complementary DNA; F, female; LB, likely benign; Lyso-Gb3, globotriaosylsphingosine; LP, likely pathogenic; M, male; NR, not recorded; P, pathogenic.

**Table 4:** Kidney Characteristics and Mainz Severity Score Index of the Fabry Disease Cohort.

Case	Sex	Age Group	Baseline						Last Follow-Up						HT ^c^	RAAS Blocker ^c^	KRT	MSSI
			SCr (mg/dL)	eGFR	CKD	UACR ^a^	UPCR ^a^	Dip ^b^	SCr	eGFR	CKD	UACR ^a^	UPCR ^a^	Dip ^b^				
1	F	A	0.63	114	G1	1650	2570	3+	0.64	111	G1	475	1722	3+	Yes	Yes	No	8
2	F	P	0.49	144	G1	11	146	Neg	0.52	158	G1	NR	162	Neg	No	No	No	NA
3	M	P	0.67	177	G1	NR	NR	Neg	0.81	156	G1	NR	NR	1+	No	No	No	NA
4	F	P	0.64	134	G1	NR	NR	3+	0.69	195	G1	NR	NR	1+	No	No	No	NA
5	M	A	1.26	63	G2	NR	NR	1+	1.02	81	G2	NR	NR	2+	Yes	Yes	No	14
6	F	P	0.53	152	G1	167	548	1+	0.56	152	G1	NR	NR	3+	No	No	No	NA
7	M	P	0.71	131	G1	NR	NR	Neg	0.61	212	G1	NR	NR	Neg	No	No	No	NA
8	F	A	0.67	118	G1	29	164	Neg	0.61	121	G1	NR	NR	Neg	No	No	No	3
9	M	A	0.93	113	G1	7	72	Neg	1.01	97	G1	5	40	Neg	No	No	No	9
10	M	A	0.83	126	G1	2	14	Neg	0.83	120	G1	2	55	Neg	No	No	No	3
11	M	A	1.36	70	G2	52	1325	2+	2.87	14	G5	1925	2796	3+	Yes	Yes	Yes	39
12	F	A	1.11	55	G3	4259	5673	3+	1.05	57	G3	NR	NR	4+	Yes	Yes	No	34
13	F	P	0.45	161	G1	32	136	Neg	0.43	194	G1	275	467	2+	Yes	Yes	No	NA
14	F	P	0.41	156	G1	26	188	Neg	0.58	127	G1	4	84	Neg	No	No	No	NA
15	M	A	0.70	110	G1	NR	338	1+	0.67	115	G1	NR	NR	1+	Yes	Yes	No	16
16	F	A	0.68	105	G1	NR	252	1+	0.47	110	G1	45	285	1+	No	No	No	15

^a^ UACR, urine albumin-to-creatinine ratio; UPCR, urine protein-to-creatinine ratio; both are expressed in mg/g. ^b^ Dip, dipstick proteinuria; Neg, negative; 1+ to 4+, semiquantitative grades. ^c^ HT and RAAS blocker use indicate status at the last follow-up. A, adult; CKD, chronic kidney disease; eGFR (mL/min/1,73 m^2^), estimated glomerular filtration rate; F, female; HT, hypertension; M, male; MSSI, Mainz Severity Score Index; NA, not applicable; NR, not recorded; P, pediatric; RAAS, renin–angiotensin–aldosterone system; KRT, kidney replacement therapy; SCr, serum creatinine.

## Data Availability

De-identified data supporting the findings may be requested from the corresponding author, subject to ethics approval and institutional data-sharing requirements.
